# Harnessing neurovascular interaction to guide axon growth

**DOI:** 10.1038/s41598-019-38558-y

**Published:** 2019-02-18

**Authors:** Paul P. Partyka, Ying Jin, Julien Bouyer, Angelica DaSilva, George A. Godsey, Robert G. Nagele, Itzhak Fischer, Peter A. Galie

**Affiliations:** 1 Rowan University, Department of Biomedical Engineering, Glassboro, New Jersey 08028 USA; 2 Drexel University College of Medicine, Department of Neurobiology and Anatomy, Philadelphia, Pennsylvania 19129 USA; 3 Rowan University, School of Biomedical Sciences, Stratford, New Jersey 08084 USA; 4 Rowan University, School of Osteopathic Medicine, Stratford, New Jersey 08084 USA

## Abstract

Regulating the intrinsic interactions between blood vessels and nerve cells has the potential to enhance repair and regeneration of the central nervous system. Here, we evaluate the efficacy of aligned microvessels to induce and control directional axon growth from neural progenitor cells *in vitro* and host axons in a rat spinal cord injury model. Interstitial fluid flow aligned microvessels generated from co-cultures of cerebral-derived endothelial cells and pericytes in a three-dimensional scaffold. The endothelial barrier function was evaluated by immunostaining for tight junction proteins and quantifying the permeability coefficient (~10^−7^ cm/s). Addition of neural progenitor cells to the co-culture resulted in the extension of Tuj-positive axons in the direction of the microvessels. To validate these findings *in vivo*, scaffolds were transplanted into an acute spinal cord hemisection injury with microvessels aligned with the rostral-caudal direction. At three weeks post-surgery, sagittal sections indicated close alignment between the host axons and the transplanted microvessels. Overall, this work demonstrates the efficacy of exploiting neurovascular interaction to direct axon growth in the injured spinal cord and the potential to use this strategy to facilitate central nervous system regeneration.

## Introduction

Beginning in the early stages of development, vascular and neural networks are intimately linked in the central nervous system (CNS). The developing brain and spinal cord lack resident vascular precursor cells^1^, thus angiogenesis from the perineural vascular plexus is required to vascularize the neural tube^2–4^. Consequently, vascular and neural systems are patterned in parallel and exhibit spatial proximity and alignment throughout the central and peripheral nervous systems^5–10^. In addition to their structural association, the function of these two systems is also closely intertwined. Neural activity is associated with localized increases in cerebral blood flow^11^, and cerebral vasculature combines with neural progenitor cells to form a “neurovascular niche” that supports neurogenesis in adulthood^12^. The close relationship between vascular and neural systems suggests their interaction may be exploited to regenerate and repair the CNS following injury and disease.

In particular, one potential strategy to capitalize on neurovascular interaction involves using recent advances in vascular patterning^13–20^ to control the orientation of regenerating axons. The spinal cord provides an excellent platform to investigate vascular-guided axon growth, since axon tracts run primarily in one direction along the rostral-caudal axis and the inflammatory environment following injury inhibits spontaneous neuroregeneration^21,22^. Conduits delivered to the site of a spinal cord injury (SCI) that are permissive to regenerating axons^23–26^ provide a means to interrogate the effect of vascular orientation on the direction of axon growth.

In this study, we describe the fabrication and alignment of microvessels within a conduit suitable for transplantation into the damaged spinal cord. Immunofluorescence and permeability assays verify that the microvessels exhibit a functional blood-spinal cord barrier (BSCB) containing tight junctions, which is characteristic of spinal cord vasculature and thus necessary for eventual incorporation into the local vascular bed. The effect of microvessel orientation on directional axon growth is first evaluated by seeding neural progenitor cells within the scaffold *in vitro*. Transplantation of the scaffolds into a cervical hemisection SCI rat model then provides validation of the efficacy of vascular-guided axon growth *in vivo*.

## Results

### 3D *in vitro* microvessel formation and alignment

**Figure 1:**
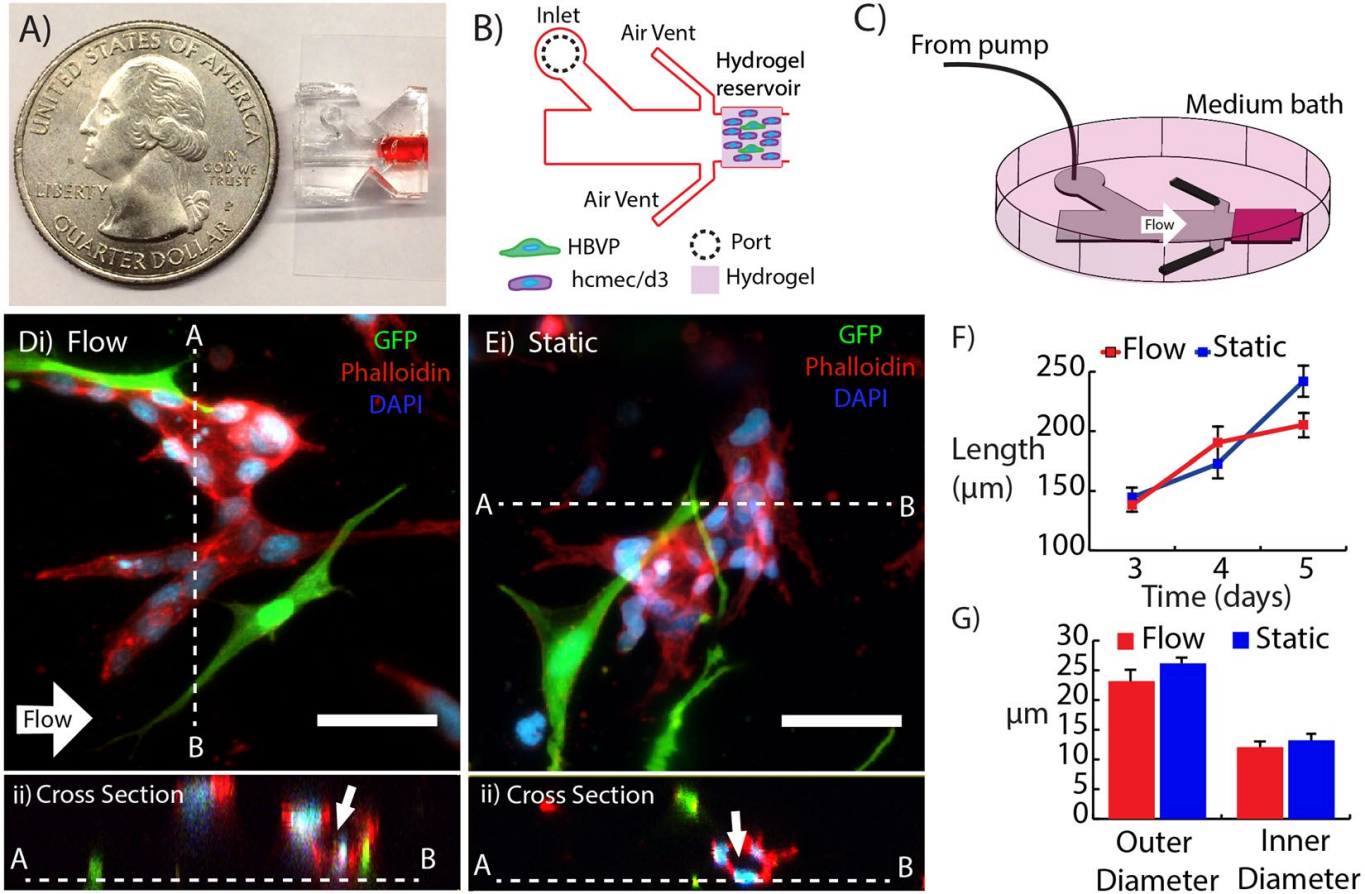
3D *in vitro* microvessel formation. Microfluidic device (**A**) photograph, (**B**) schematic, and (**C**) setup for flow. (**D**) Microvessel exposed to interstitial fluid flow (direction denoted by white arrow) with cross section (white dashed line) showing lumen (ii). (**Ei**) Microvessel from static control with cross section showing lumen (arrow) (ii). (**F**,**G**) Microvessel length and diameter as a function of time. GFP hBVP (green), Phalloidin-Texas Red (red), and DAPI (blue). Scale bars, 50 μm.

Composite hydrogels consisting of 5-mg/mL type 1 collagen and 3-mg/mL hyaluronan were polymerized within a perfusable microfluidic device (Fig. 1A,B). The devices were connected to a syringe pump and submersed in a well plate filled with culture medium (Fig. 1C). After 2–3 days in culture, the co-culture of pericytes and cerebral-derived endothelial cells formed multi-cellular structures within the hydrogel in both perfused (3 µm/s interstitial flow velocity) and static conditions (Fig. 1D,i;E,i). Between days 3 and 5, these multi-cellular structures displayed continuous lumens visible during confocal imaging (Fig. 1D,ii,E,ii), and are hereafter referred to as microvessels. Measurements indicated that application of interstitial fluid flow did not have a significant effect on either the length (Fig. 1F) or diameter (Fig. 1G) of the microvessels. After five days, the vessel length extended to approximately 200–250 µm. The lumen diameter ranged from 12–13 µm, which is within the range of physiological values for microvasculature^27^.

**Figure 2:**
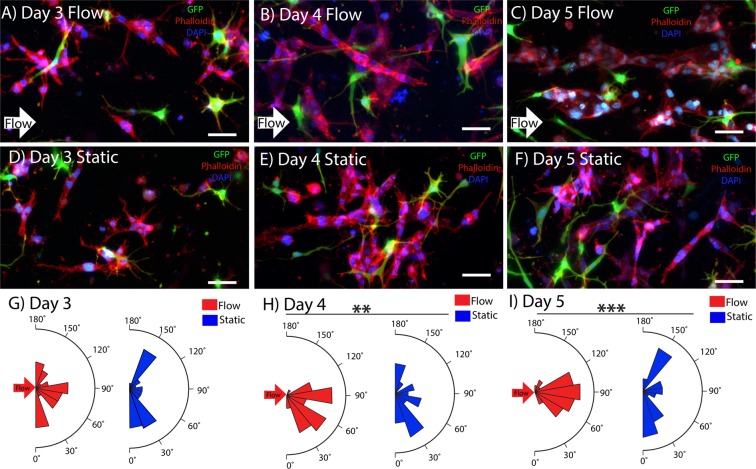
3D Microvessel alignment with interstitial fluid flow. Microvessel alignment with flow (direction denoted by white arrow) at day 3 (**A**), day 4 (**B**), and day 5 (**C**). Microvessel orientation in static conditions for day 3 (**D**), day 4 (**E**), and day 5 (**F**). Microvessel alignment plots over time for day 3 (**G**), day 4 (**H**), and day 5 (**I**). GFP hBVP (green), Phalloidin-Texas Red (red), and DAPI (blue). Scale bars, 50 μm. Data are presented as mean ± s.e.m. **P < 0.01, ***P < 0.001; statistical significance was calculated using ANOVA and post-hoc Tukey’s HSD test. Alignment values (n = 30) are from individual hydrogel samples at each time and condition (flow or static).

Having demonstrated the ability to create capillary-scale microvessels within the scaffold, experiments were conducted to determine the effect of interstitial flow on alignment. We observed that application of interstitial fluid flow had a significant effect on vascular alignment. Figure 2A–C shows microvessel orientation within hydrogels exposed to flow between days 3 and day 5, and Fig. 2D–F shows vessels exposed to static conditions over that same timespan. Microvessels exposed to flow had significantly higher levels of alignment in the flow direction compared to static conditions at days 4 and 5. Quantification of the angle between the long axis of the microvessel and the flow direction (Fig. 2G–I) also indicated that alignment of vessels exposed to flow was significantly higher at day 5 compared to day 3 (no significant difference was observed between day-4 flow and day-3 flow conditions).

### Disruption of flow-mediated alignment by disrupting cd44

**Figure 3:**
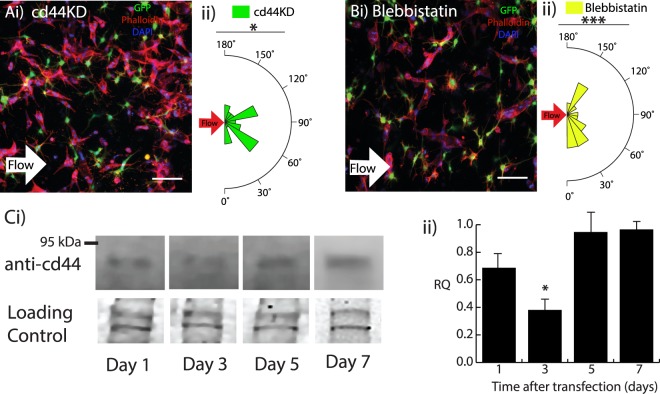
Disruption of 3D microvessel alignment. (**A**i) Day 5 scaffold exposed to interstitial fluid flow (direction denoted by white arrow) with hCMEC/D3 cd44KD cells and microvessel alignment plot (ii). (**Bi**) Day 5 hydrogel exposed to interstitial fluid flow with 0.5 μM blebbistatin and microvessel alignment plot (ii). GFP hBVP (green), Phalloidin-Texas Red (red), and DAPI (blue). Scale bars, 100 μm. (**C,i**) Western blot of cd44 protein expression levels from day 1 after transfection to day 7 and (ii) bar graph showing cd44 protein expression levels as relative intensity (RQ) normalized to day 7 values. Coomassie blue was used to control for gel loading. Data are presented as mean ± s.e.m. *P < 0.05, ***P < 0.001; statistical significance was calculated using Welch Two Sample t-test. Alignment values (n = 30) are from single samples per condition.

In order to provide additional control over vascular alignment, experiments were conducted to identify the mechanisms underlying flow-induced alignment. Perfusion with interstitial fluid flow exerts a shear stress on the endothelial cells, so we investigated potential mechanosensors that could mediate the morphological response. Given that hyaluronan is a primary component of the scaffold and that previous studies have identified its primary receptor, cd44, as a contributor to shear stress mechanotransduction^28^, siRNA was used to knockdown cd44 expression levels in the endothelial cells. Knockdown of cd44 (cd44KD) resulted in disruption of the alignment of microvessels exposed to interstitial fluid flow for five days (Fig. 3A,i). Figure 3A,ii provides quantification of the alignment. To further implicate cd44, which is known to act through Rho/ROCK signaling^29,30^, the effect of low concentrations of blebbistatin (0.5 μM) on alignment was evaluated. Confocal imaging showed disruption of alignment after attenuating myosin II-mediated contractility (Fig. 3B,i). Figure 3B,ii indicates that blebbistatin treatment also resulted in significant decreases in microvessel alignment in response to interstitial fluid flow. Neither the cd44KD nor the blebbistatin conditions affected the microvessel length (Supplemental Fig. 1A,i) compared to normal conditions perfused with flow. However, the microvessel outer diameter for the blebbistatin condition and the inner diameter for both the cd44KD and blebbistatin conditions were significantly lower (Supplemental Fig. 1B,ii). The dynamics and validation of the cd44 knockdown were evaluated using Western blotting (Fig. 3C,i), which indicated that the highest knockdown (~40% of Day 7 value) occurred at day 3 (Fig. 3C,ii), which is consistent with the finding that alignment occurs between days 3 and 5.

### Blood-spinal cord barrier (BSCB) evaluation

**Figure 4:**
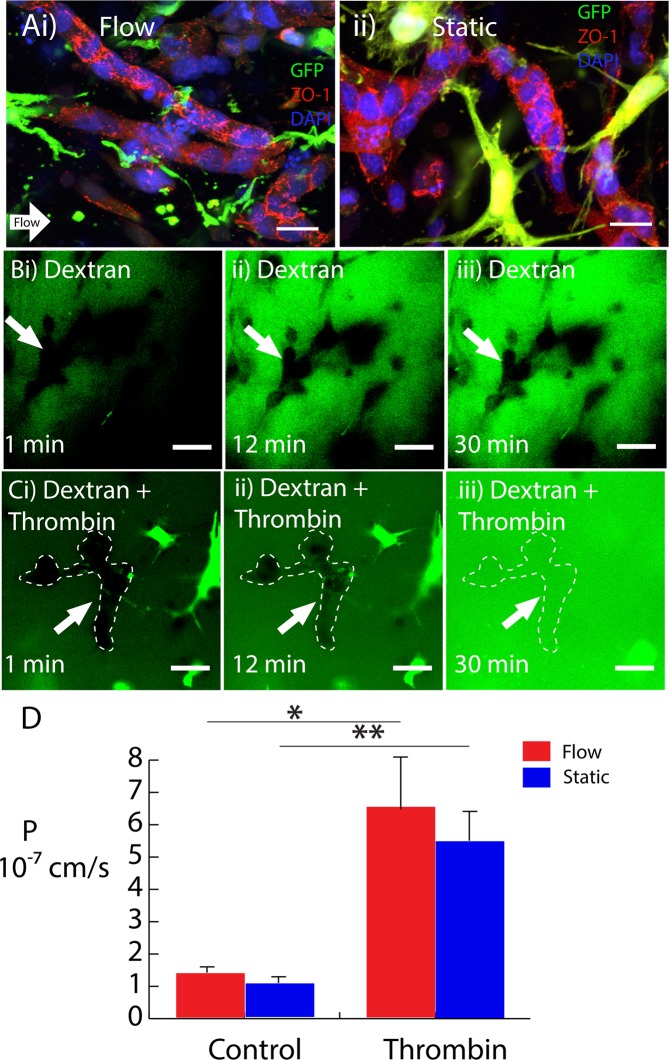
Blood-spinal cord barrier evaluation. (**A**) Day 5 scaffold exhibiting ZO-1 (red) localization to the cell-cell junctions, pericytes (GFP), and nuclei (DAPI) for flow (i) and static (ii) conditions. (**B**) 4-kDa FITC-dextran permeability test for static condition at (i) 1 min, (ii) 12 min, and (iii) 30 min. (**C**) 4-kDa FITC-dextran perfused with thrombin for static condition at (i) 1 min, (ii) 12 min, and (iii) 30 min. White dashed lines show microvessel contour. (**D**) Permeability values. Scale bars, 20 μm (**A**) and 50 μm (**B**,**C**). Data are presented as mean ± s.e.m. *P < 0.05, **P < 0.01; statistical significance was calculated using Welch Two Sample t-test.

The microvessel barrier integrity was evaluated using both immunostaining of tight junction proteins and a dextran diffusion assay developed for this study. Figure 4A demonstrates that the tight junction scaffolding protein, zonula-occludin-1 (ZO-1), localized to the cell-cell junctions of endothelial cells within the microvessels. The ZO-1 staining localized to the cell-cell junctions regardless of whether the microvessels were cultured under perfused or static conditions. To provide a more quantitative measure of the barrier integrity of the vessels, 4-kDa FITC-dextran was perfused through the bulk of the hydrogels and high scan-rate confocal microscopy was used to measure dextran exclusion from the vessel lumens (Fig. 4B). During the course of the 30-minute perfusion, dextran was mostly excluded from the microvessel lumen (Fig. 4B,i–iii). To provide a negative control, FITC-dextran was perfused with 10 U/mL thrombin (Fig. 4C) to disrupt barrier integrity. The thrombin eventually resulted in complete saturation of the vessel lumen with FITC-dextran during the same 30-minute span (Fig. 4C,i–iii). Figure 4D shows the permeability values calculated from these measurements, using the dextran fluorescence intensity as an indication of concentration. The measured values had magnitudes consistent with *in vivo* measurements^31^. As expected from the results of the immunofluorescence studies, no significant difference was observed between the permeability of microvessels exposed to perfused and static conditions.

### Patterned microvessels guide axons from neural progenitor cells (NPCs)

**Figure 5:**
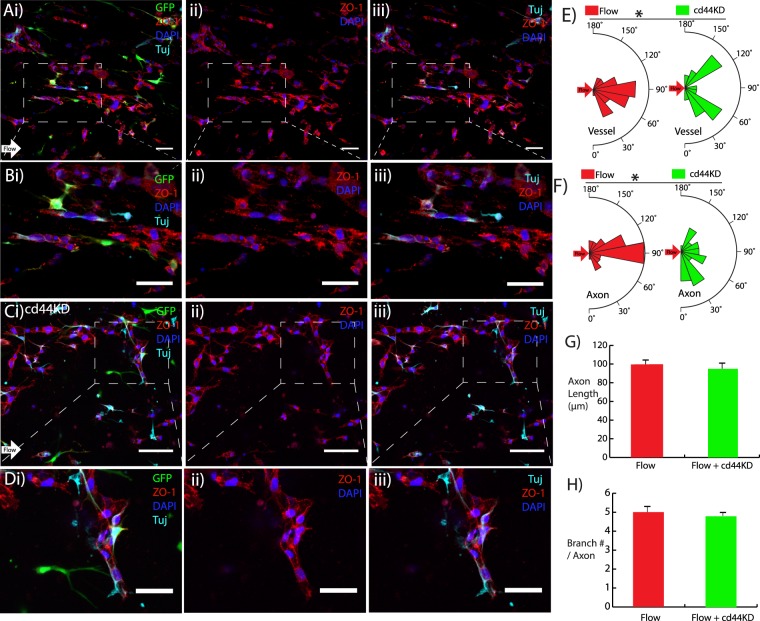
Patterned microvessels guide axons from NPCs *in vitro* (**Ai**) Day 4 axon alignment with microvessels (direction denoted by white arrow), (ii) ZO-1 tight junction stain (red), and (iii) axons labeled with Tuj (cyan) for flow condition. (**B**) Higher magnification images from A. (**Ci**) Day 4 cd44KD with flow condition, (ii) ZO-1 tight junction stain (red), and iii) Tuj-positive axons (cyan), GFP hBVP (green), DAPI (blue). (**D**) Higher magnification images from C. (**E**,**F**) Axon and vessel alignment quantification for flow and cd44KD conditions. (**G**,**H**) Axon length and branch number for both flow and cd44KD conditions. Scale bars, 50 μm. Data are presented as mean ± s.e.m. *P < 0.05 compared to untreated flow condition. Alignment values (n = 30), axon length values (n = 15), and branch number/axon values (n = 5) are from single hydrogel samples per condition.

To evaluate the ability of patterned microvessels to align axon growth along a prescribed direction, an initial *in vitro* experiment was conducted by seeding NPCs within the scaffold containing pericytes and cerebral-derived endothelial cells. Interstitial fluid flow containing neurotrophin-3 (NT-3) was applied to align the microvessels in the intended direction of axon growth and stimulate NPC differentiation to a neuronal phenotype. After four days of perfusion, Tuj-positive axons from the NPCs extended along the flow direction, parallel to the long axis of aligned microvessels. Figure 5A provides a low-magnification image of the aligned vessels labeled with ZO-1 adjacent to Tuj-positive axons extending from differentiated NPCs. Figure 5B focuses on one segment indicating that the axons aligned with the microvessels in the direction of perfusion. In Fig. 5C, hydrogels seeded with cd44 knockdown endothelial cells were also exposed to interstitial fluid flow. Due to the disruption of cd44-mediated signaling, the flow-induced alignment was negated and the Tuj-positive axons no longer oriented in the direction of perfusion. Figure 5D provides a higher magnification image of this condition, indicating that the axons still exhibited close proximity to the microvessels, further suggesting that microvessels dictate axon orientation regardless of alignment. Quantification of both vessel and axon orientation (Fig. 5E,F) indicated significantly higher alignment in the direction of flow in cells without cd44 knockdown. Additionally, the length of Tuj-positive axons and the number of branches per axon were not significantly affected by cd44 knockdown (Fig. 5G,H).

### Patterned microvessels guide host axons in an acute spinal cord injury (SCI) model

**Figure 6:**
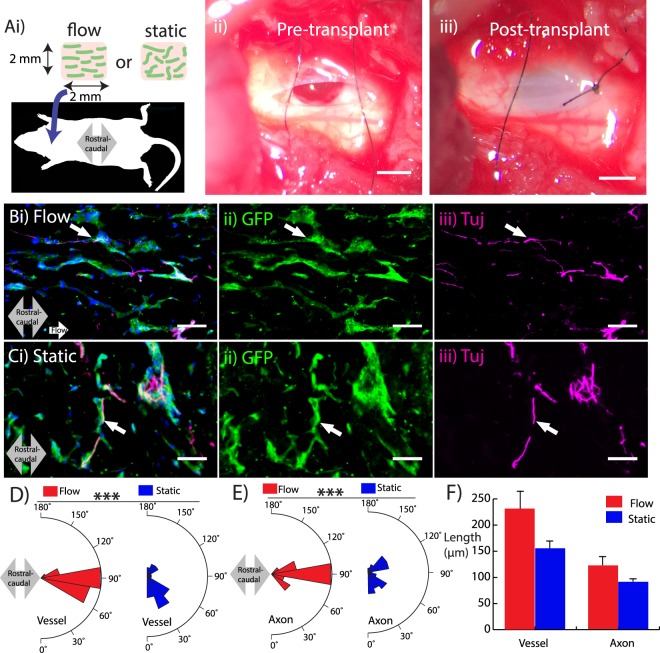
Axon guidance at the site of a cervical spinal cord injury in a rat model. (**Ai**) Schematic illustrating transplantation of scaffold into a C-4 hemisection. The injury cavity is shown prior to (ii) and immediately following (iii) transplantation. (**Bi**) Scaffold conditioned with flow exhibits viable GFP-labeled microvessels (green) (ii) and alignment of host axons (magenta) infiltrating the scaffold in the rostral-caudal direction (grey arrow). (**C**) Scaffold conditioned in static conditions showing disrupted alignment of both microvessels (ii) and host axons (iii). (**D**–**F**) Microvessel and axon plots showing alignment (**D**,**E**) and length (**F**). Scale bars, 1 mm (Aii,Aiii) and 50 μm (**B**,**C**). Data are presented as mean ± s.e.m. ***P < 0.001; statistical significance was calculated using Welch Two Sample t-test. White arrows denote proximity of axons with microvessels. Microvessel alignment values (n = 30), axon alignment values (n = 30), microvessel length values (n = 15), and axon length values (n = 15) are from single hydrogel samples per condition.

Having demonstrated that aligned microvessels guide the direction of axons *in vitro*, an *in vivo* study was conducted to evaluate the efficacy of vascular-guided axon growth in a rat model of SCI. Cerebral-derived endothelial cells and pericytes were labeled with GFP to facilitate tracking after transplantation. An initial attempt with a scaffold containing 5 mg/mL collagen yielded little to no axon infiltration (Supplemental Fig. 2). Therefore, the scaffold composition used for this study consisted of 2 mg/mL collagen, 3 mg/mL hyaluronan, and 1 mg/mL Matrigel. These scaffolds were exposed to either flow or static conditions and delivered into an acute cervical hemisection injury, with the flow direction aligned with the rostral-caudal axis of the cord (Fig. 6A,i–iii). Three weeks after transplantation, immunohistochemistry was used to evaluate the viability and alignment of the transplanted microvessels as well as the presence and direction of host axons infiltrating the scaffold. Supplemental Videos 1 (10x magnification) and 2 (20x magnification) show a confocal stack of several histological sections, indicating the incorporation of the transplanted scaffold. The microvessels aligned with flow remained aligned in the rostral-caudal axis after 3 weeks (Fig. 6B,i–ii). Additionally, Fig. 6B,iii shows that Tuj-positive host axons infiltrated the scaffold along microvessels in the rostral-caudal direction. (Supplemental Fig. 3 shows positive CGRP staining indicative of regenerating axons).

In contrast, microvessels in the scaffold exposed to static conditions exhibited random alignment (Fig. 6C,i–ii) after transplantation. Though the scaffold instigated axon ingrowth, there was no significant alignment of the infiltrating axons with the rostral-caudal direction (Fig. 6C,iii). In both cases, the axons closely interacted with the microvessels with a high degree of spatial proximity, similar to the *in vitro* NPC experiments. Quantification indicated a significant increase in alignment with the rostral-caudal direction in the perfused scaffolds compared to static controls (Fig. 6D,E), though there was no difference in length of the microvessels and axons between the flow and static conditions (Fig. 6F). These results underscore the close interaction between vasculature and neural cells, and suggest that patterning microvessels with interstitial flow can be used to dictate the orientation of axon growth *in vivo*.

## Discussion

Exploiting the intrinsic interaction between neural and vascular cells holds great potential for directing axon growth in regenerative applications designed for repair and restoring connectivity in the CNS. In this study, we used interstitial fluid flow to align microvessels exhibiting BSCB-integrity within transplantable scaffolds, and then investigated the ability of these vessels to dictate axon orientation. Our results demonstrate that microvessels guide axon growth from both neural progenitors *in vitro* and infiltrating neurites *in vivo*. Blocking flow-mediated vascular alignment through the cd44 receptor verified that the direction of axon growth from neural progenitor cells is determined by the orientation of the vessels and not a flow-mediated effect. A proof-of-concept transplantation study in a hemisection spinal cord injury model corroborated the *in vitro* results by demonstrating that axons infiltrating the scaffold aligned with the patterned microvasculature. Determining that aligned microvessels guide the direction of axon growth has important implications for regenerative approaches in the CNS, specifically in the spinal cord where the ascending and descending neural tracts align primarily along the rostral-caudal axis and the challenge is not only to promote regeneration, but also to provide directional cues.

In this study, we observed that seeding a co-culture of vascular pericytes and cerebral microvascular endothelial cells within a collagen and hyaluronan composite scaffold spontaneously formed microvessels. After several days in culture, the microvessels exhibited tight junctions and low permeability values characteristic of the BSCB. The barrier formed in microvessels exposed to both perfused and static conditions. This result appears to contradict our previous finding in a 3D blood-brain barrier model that tight junction formation requires application of 0.7 dyn/cm^2^ of fluid shear stress to the lumen^32^. However, the mode and magnitude of shear stress applied to endothelial cells by bulk perfusion differ from that exerted by luminal flow, and likely changes as the cells form microvessels. Moreover, the geometry of our previous blood-brain barrier model features a diameter of 180-μm, which is an order of magnitude greater than the ~10 μm-diameter microvessels presented here. Thus, the disparity regarding the role of fluid shear stress suggests that vessel diameter affects barrier function. A recent study implicating the radius of curvature of a substrate as an important regulator of cell signaling and morphology^33^ lends credence to vessel diameter regulating barrier formation, though further investigation in this model is warranted.

Although BSCB integrity is required for functional incorporation into the host vascular bed, our results do not interrogate whether barrier function is required to guide axon growth. A previous study focused on the peripheral nervous system suggests that barrier formation is not required for axonal guidance: non-CNS endothelial cells stimulated and directed the growth of axons from a dorsal root ganglion through VEGF-mediated paracrine signaling^34^. VEGF is a growth factor associated with the growth of both the vascular and neural systems in the central nervous system^2^, and may contribute to the mechanism underlying the vascular-guided axon growth observed here regardless of the barrier integrity of the microvessels. However, inspection of the immunocytochemistry data indicates close proximity between axons and microvessels, which suggests a juxtacrine signaling mechanism. The basal lamina, a crucial component of the neurovascular unit^35^, may contribute to this mechanism. Given that previous studies have found collagen IV and laminin, major components of the endothelial basal lamina, induce and guide axon growth^36,37^, secretion of these proteins by the cerebral endothelial cells may mediate neurovascular interaction.

Our results demonstrate that patterned vasculature induces axon infiltration from the host and guides their growth, indicating the potential of this scaffold to support axon connectivity and ultimately functional benefits in the damaged spinal cord. SCI creates a complex and inflammatory microenvironment that inhibits axon growth and impedes spontaneous regeneration^21^. The injury environment, which lacks proper blood supply, also presents a challenge to the viability of cells transplanted into the injury site in regenerative applications^38^. Previous studies indicate that transplanting conduits with pro-angiogenic factors^39–41^ improves neuroregeneration, suggesting the benefit of delivering oxygen and nutrients through vascularization. Furthermore, an imaging analysis of vascular and axonal networks in the injured spinal cord found that axon growth rate was substantially increased in close proximity to vasculature^42^. Therefore, aligned microvessels have the dual benefit of providing the basis for a vascular bed within the scaffold to promote cell survival and directing the growth of regenerating axons. Future studies will evaluate the functional benefit resulting from delivery of this multifunctional treatment strategy in various models of CNS injury.

## Methods

### Microvasculature fabrication

Microvessels were fabricated in collagen and hylauronan (HA) composite hydrogels polymerized inside polydimethylsiloxane (PDMS)-based microfluidic devices fabricated using soft lithography^43^. p20–p23 human cerebral microvascular endothelial cells (hCMEC/D3) and p7–p15 GFP-labeled human brain vascular pericytes (hBVP) (Neuromics) were seeded in the hydrogels at densities of 2 M/mL and 0.4 M/mL, respectively. These ratios were obtained from a previous study that used co-cultures of human blood outgrowth endothelial cells and human pericytes to form microvasculature networks^13^. hCMEC/D3 were cultured in Endothelial Cell Basal Medium (PromoCell) supplemented with 5 µg/mL ascorbic acid (Sigma), 1 ng/mL hBFGF (Sigma), 1/100 chemically defined lipid concentrate (Thermo Fisher), 5% fetal bovine serum (VWR Life Science), 10 mM HEPES (Quality Biological), 1.4 µM hydrocortisone (Sigma), and 1% penicillin-streptomycin (Corning). hBVP were cultured in DMEM (Corning) supplemented with 10% FBS, 1% penicillin-steptomycin, and 1X MEM Amino Acid Solution (Thermo Fisher). Final hydrogel formulation concentrations consisted of 3 mg/mL HA (Sigma), 5 mg/mL collagen type I (MP Biomedical), and 0.85–1 mg/mL Matrigel (Corning). These reagents were combined with 0.1 M sodium hydroxide (NaOH) and 10x phosphate buffer solution (PBS) to facilitate polymerization and maintain physiological pH.

### Quantification of vessel length, diameter, and alignment *in vitro*

Cell-seeded hydrogels were exposed to either static conditions (control) or perfused with interstitial flow velocity of 3 µm/s for 5 days, to match previous alignment studies^44^. At each time point, gels were fixed at room temperature for 20 minutes with 4% paraformaldehyde, permeabilized with 0.2% Triton X-100 for 20 minutes, and then stained with 1:100 Texas Red-phalloidin (Biotium) and 1:500 DAPI. Samples were imaged with a laser scanning confocal microscope (Nikon C2), which was used to generate z-stacks that were then analyzed with Nikon Elements Analysis software. Microvessels were identified by the presence of a distinct, three-dimensional lumen. We then measured the length of the microvessels by comparing the maximum projection and 3D volume views to determine the beginning and end microvessel positions. Microvessel alignment was measured using the reference angle tool with the reference angle being the direction of interstitial flow.

### cd44 knockdown and quantification

siRNA (Santa Cruz Biotechnology) targeting cd44 was used to knock down its gene expression in the HCMEC/D3 cells. Cells were plated in six well plates and grown until confluent. A solution of 8 µL siRNA duplex, 8 µL siRNA transfection reagent, and 1 mL of siRNA transfection medium was introduced to the cells in each well. The cells were incubated for 5 hours at 37 °C after which 1 mL of EGM containing 2% penicillin/streptomycin and 10% fetal bovine serum was added without removing the transfection mixture. The cells were incubated for an additional 18 hours after which they were used for experiments. To verify knockdown, cellular protein was isolated using digestion in sample buffer with reducing agent, and separated using SDS-PAGE. Following transferm PVDF blots were incubated with an anti-cd44 primary (1:50) and horseradish peroxidase-conjugated secondary (1:4000) within an iBind Flex Western Device (Thermo Scientific). Blots were imaged in a chemiluminescent imager. To provide a loading control, gels were incubated in Coomassie Blue for 30 minutes *following the transfer* and representative bands were used to normalize blot signals.

### Blood-brain barrier verification

Microfluidic devices of day 5 samples were secured to a 22 mm × 40 mm cover slip with 2–5 µL of super glue and transferred to the stage of the confocal microscope. Microvascular structures within the hydrogel were located using the brightfield prior to perfusion with 4-kDa FITC-dextran (1:250) at a flow rate of 2 μL/min. Individual confocal slices were captured every minute at approximately 8–10 different sections within the hydrogel. Images were processed using ImageJ software where the permeability was calculated using a modified version of the method described by Adamson^45^. To accommodate for interstitial dextran perfusion instead of intraluminal, the change in fluorescent intensity was measured inside the lumen and the maximum intensity was measured outside the lumen. To perform a negative control, 10 U/mL thrombin (Calbiochem) was added to the 4-kDa dextran solution perfusing the samples for 30 minutes.

To visualize the presence of tight junctions, samples were blocked in 3% BSA for 30 minutes at room temperature. Then the samples were incubated at 4 °C overnight with 1:250 primary antibody ZO-1 (Cell Signaling). The next day hydrogels were incubated at 37 °C with 1:500 secondary antibody Cy-3 for 1 hour and visualized with the confocal microscope.

### Seeding hydrogels with NPC

Rat-derived E14 neural progenitor cells (NPC) were seeded within the co-culture of the GFP-labeled human brain vascular pericytes (hBVP) and human cerebral microvascular endothelial cells (hCMEC/D3) at a ratio of 1:2 with hCMEC/D3. NPC isolation and culture was performed at Drexel University and is detailed in the following reference^46^. Hydrogels were perfused with complete endothelial growth media supplemented with 20 µg/mL NT-3 growth factor (PeproTech) to maintain the viability of NPCs and facilitate differentiation to neuronal restricted precursors (NRP), identified by positive staining for Tuj-1. Hydrogels seeded with rat-derived neural progenitor cells (NPC) were double stained to visualize both the endothelial tight junctions and axons. All the following steps were done at room temperature. Hydrogels were blocked in 10% normal donkey serum (NDS) for 10 minutes followed by incubation with 1:500 Tuj-1 (Biolegend), 1:250 ZO-1, and 2% NDS for 2 hours. After this the hydrogels were incubated with 1:500 DyLight 650 conjugate, 1:500 Cy-3, and 2% NDS for 1 hour. Positive branches extending from each Tuj-positive were identified by subtracting the 640 nm Cy5 signal (Tuj-1) from both the 561 nm TRITC (ZO-1) and the 488 nm GFP signals. These branches were then totaled using the counter option in the Nikon Elements software and compared between flow and static samples.

### Scaffold transplantation *in vivo*

For the *in vivo* transplantation, the collagen concentration was reduced to 2.5 mg/mL to increase axon permissivity. Day 2 scaffolds (either exposed to flow or static conditions) were transplanted into acute animal hemisection spinal cord injury model by aspiration at the level of the fourth cervical vertebrae of the spinal cord 1-mm lateral of the midline from the posterior side. In total, 4 female adult Sprague-Dawley rats (225–250 g) were used. Surgeries were conducted at the Drexel University Queen Lane Medical Campus in accordance with the IACUC agreement, which was approved by the Drexel College of Medicine Institutional Review Board. Animals were administered cyclosporine three days prior to surgery and during the 3-week experiment as a means to minimize host inflammatory response. Following transplantation, animals were caged in their normal environments with normal food and water intake. Animals were sacrificed 3 weeks after transplantation and immunohistochemistry was performed to process the data using serial 8-µm sagittal sections. These sections were visualized in a confocal microscope, and stacks were concatenated to form a three-dimensional rendering of the transplant. These 3D renderings were used to measure both microvessel and axon length to account for extensions out-of-plane from single sagittal sections and to assure that the measurements were representative of their full lengths.

### Statistics

For all data, statistical significance was calculated using Welch Two Sample t-test, unless otherwise specified. Statistical significance of the microvessel alignment data was calculated using one-way ANOVA and post-hoc Tukey’s HSD tests. Statistical significance was denoted with p-values less than 0.05. Each *in vivo* condition (four animals total) is averaged from at least 5 histological sections. Figure captions contain the sample number for the *in vitro* measurements.

## Supplementary information

Supplementary Information

Supplementary Video 1

Supplementary Video 2

## Data Availability

The raw data required to reproduce these findings are available upon request of the corresponding author. The processed data required to reproduce these findings are also available upon request of the corresponding author.
