# Supplementary material for: Harnessing neurovascular interaction to guide axon growth
Source: Sci Rep. 2019 Feb 18;9:2190. doi: 10.1038/s41598-019-38558-y (PMC6379421; doi:10.1038/s41598-019-38558-y)
Supplement: Supplementary file 1 — Supplementary Information [file 41598_2019_38558_MOESM1_ESM.pdf]

# Harnessing neurovascular interaction to guide axon growth

**Authors:** Paul P. Partyka[1], Ying Jin[2], Julien Bouyer[2], Angelica DaSilva[1], George A. Godsey[3], Robert G. Nagele[3,4], Itzhak Fischer[2], Peter A. Galie[1*]

**Affiliations:**
[1] Rowan University, Department of Biomedical Engineering, Glassboro, New Jersey 08028, USA.
[2] Drexel University College of Medicine, Department of Neurobiology and Anatomy, Philadelphia, Pennsylvania 19129, USA.
[3] Rowan University, School of Biomedical Sciences, Stratford, New Jersey 08084, USA.
[4] Rowan University, School of Osteopathic Medicine, Stratford, New Jersey 08084, USA.

## SUPPLEMENTAL FIGURE LEGENDS

**Fig. s1.** Microvascular morphology in the cd44KD and blebbistatin conditions compared to controls. Ai) Comparison of microvessel length and Aii) microvessel diameter of cd44KD and blebbistatin conditions to normal day 5 perfused hydrogels. Data are presented as mean ± s.e.m. *$P<0.05$; statistical significance was calculated using Welch Two Sample t-test. Length values ($n=15$) and diameter values ($n = 5$) are from single hydrogel samples per condition.

**Fig. s2.** Transplantation of a scaffold containing 5 mg/mL collagen density. A) Low magnitude image showing a lack of axon infiltration in the location of the transplant following hemisection injury. Scale = 500 µm. B) A magnified image of the location denoted with dotted lines in panel A. White arrows show attenuated neurite infiltration labelled by both Tuj and RT97 staining. Scale = 100 µm.

**Fig. s3.** A) Regenerating axon (CGRP-positive) infiltration into the transplant three weeks after transplantation. B) Low magnitude image showing the orientation of the transplant relative to host tissue. Scale bars, 50 µm (A) and 200 µm (B).

**Fig. s4**. Non-cropped blots for Figure 3. Two blots were used to evaluate cd44 expression levels at days 1, 3, 5, and 7 after incubation with siRNA.

**Supplementary Video 1.** 3d rendering of a transplanted scaffold with aligned microvessels within the host tissue, sacrificed 3 weeks-post transplantation. Transplanted cells (green-GFP), host axons (red-anti-Tuj), and cell nuclei (blue-DAPI) are used to visualize the scaffold. The rendering is constructed from confocal stacks of serial 8 micron sections imaged with a laser scanning confocal microscope with a 10x objective.

**Supplementary Video 2.** 3d rendering of a transplanted scaffold with aligned microvessels at 20x magnification. The staining and method of measurement is the same as Supplementary Video 1.

**SUPPLEMENTAL FIGURES**

**Fig. s1**

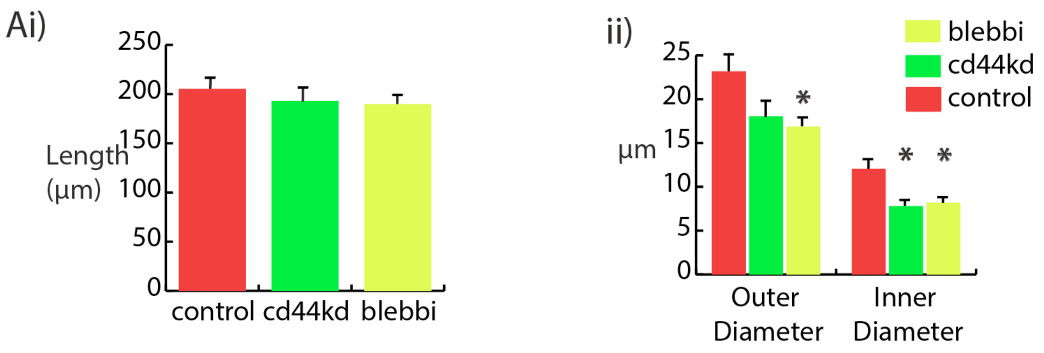

**Fig. s2**

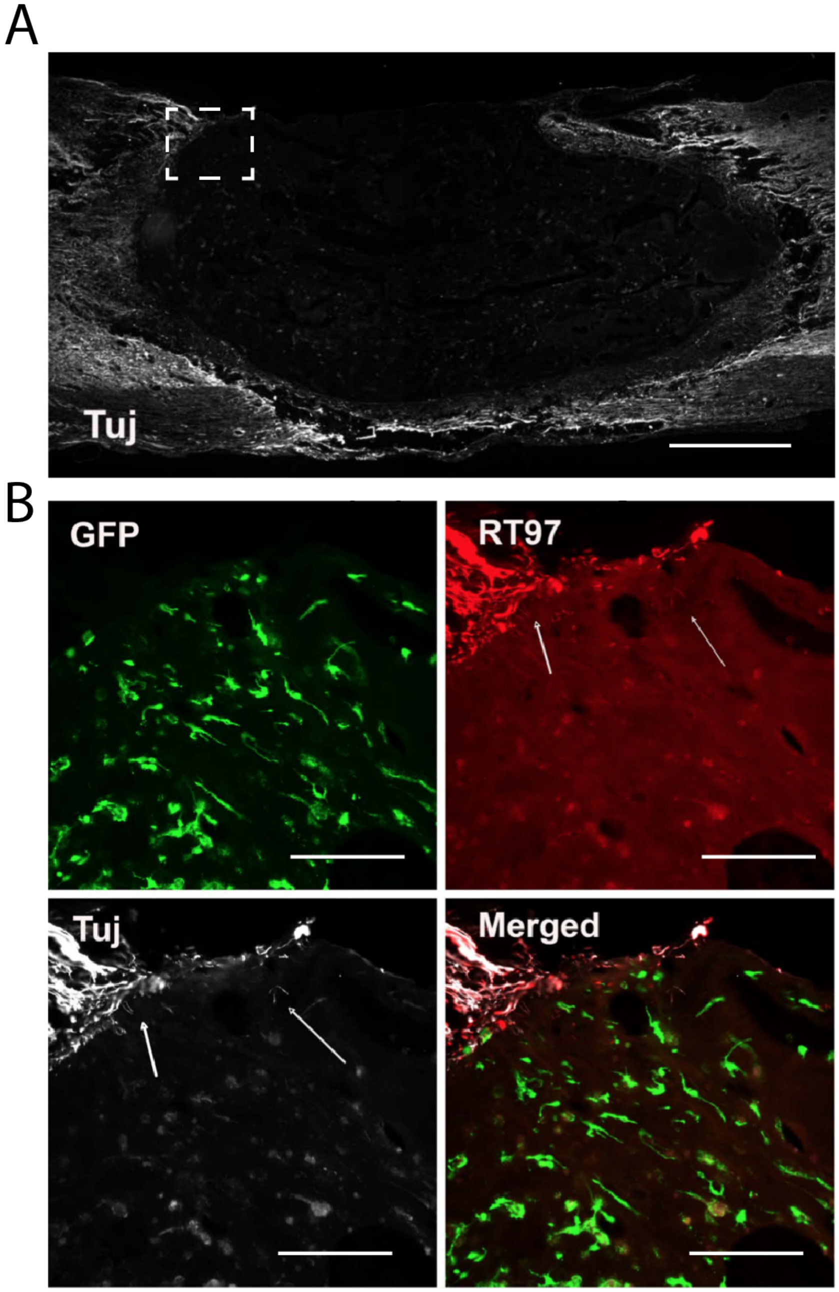

**Fig. s3**

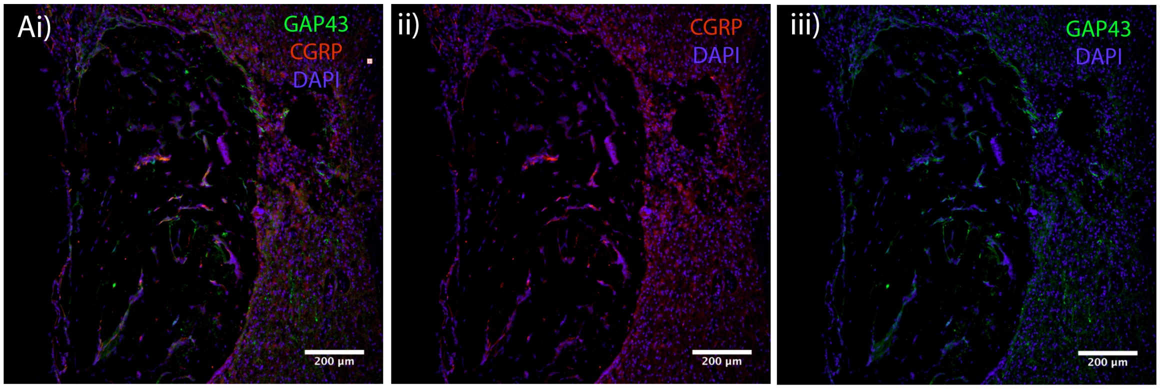


**Fig. s4**
**Blot 1 – anti-cd44**
**(representative lanes highlighted in black)**

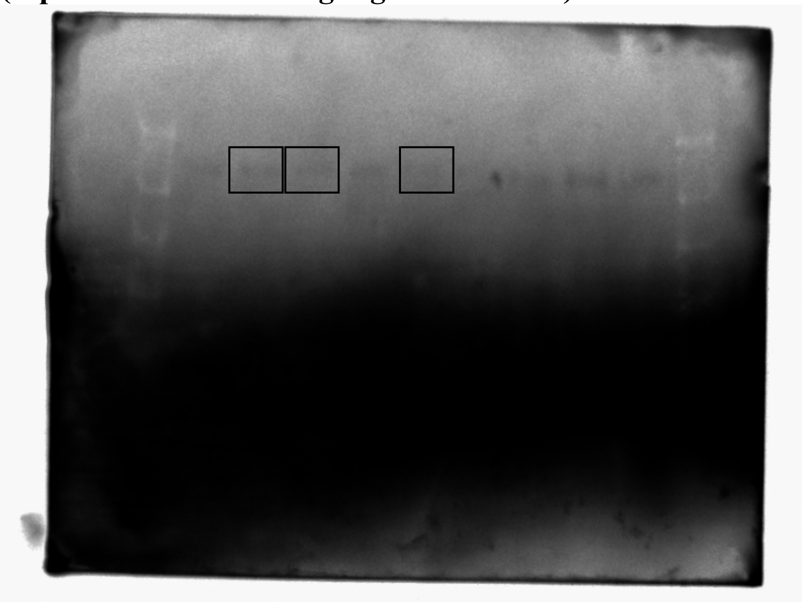

Lanes (1-9): Day1b, Day1a*, Day5b*, Day5a, Day3b*, Day3a, Day3c, Day5c, Day 7a

**Coomassie Blue stain of Gel 1**
**(representative lanes highlighted in white)**

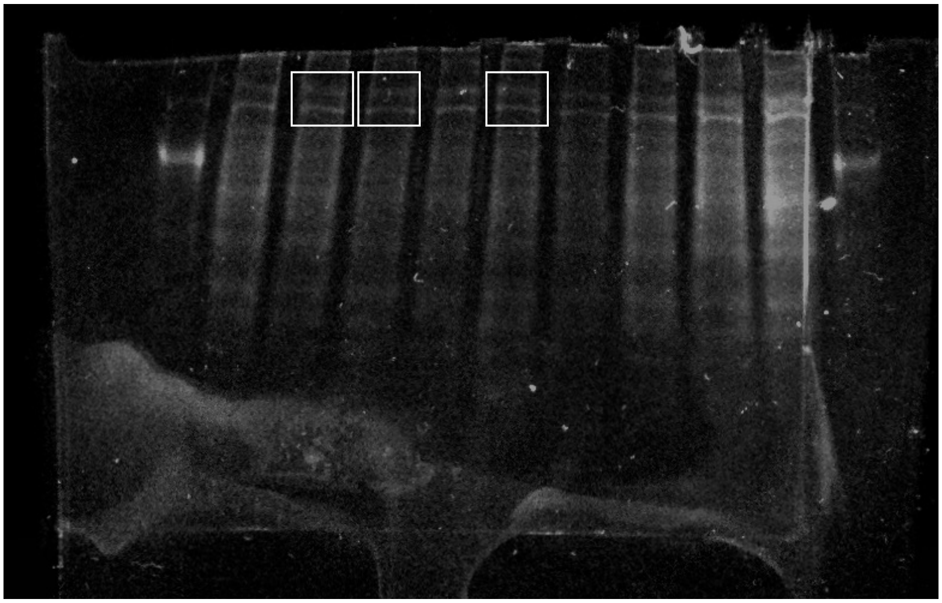

Lanes (1-9): Day1b, Day1a*, Day5b*, Day5a, Day3b*, Day3a, Day3c, Day5c, Day 7a

**Blot 2 – anti-cd44**
**(representative lanes highlighted in black)**

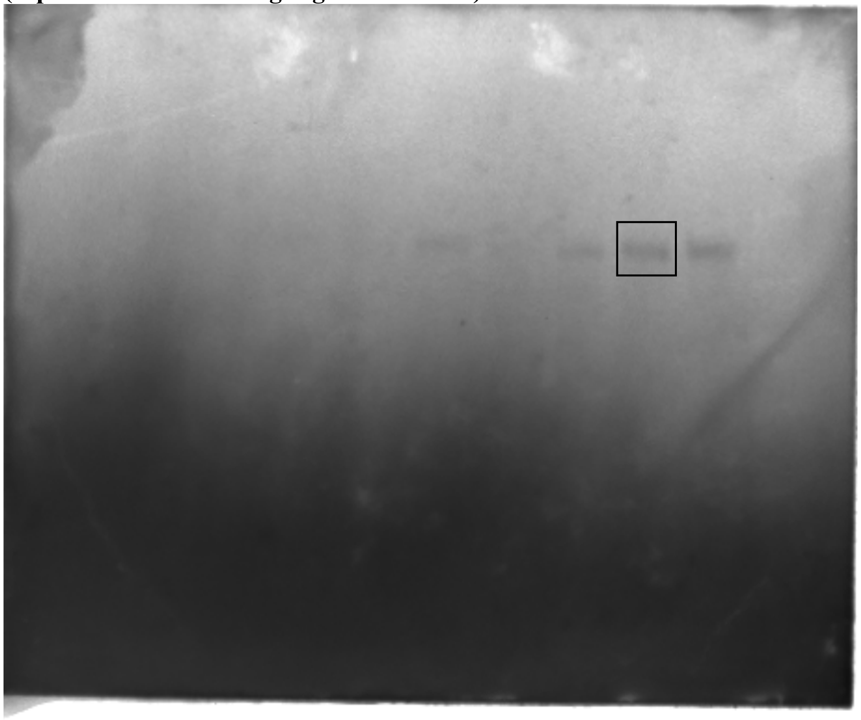

Lanes (1-7): Day3e, Day5d, Day7c, Day3d, Day1c, Day7b*, Day7a

**Coomassie Blue stain of Gel 2**
**(representative lanes highlighted in white)**

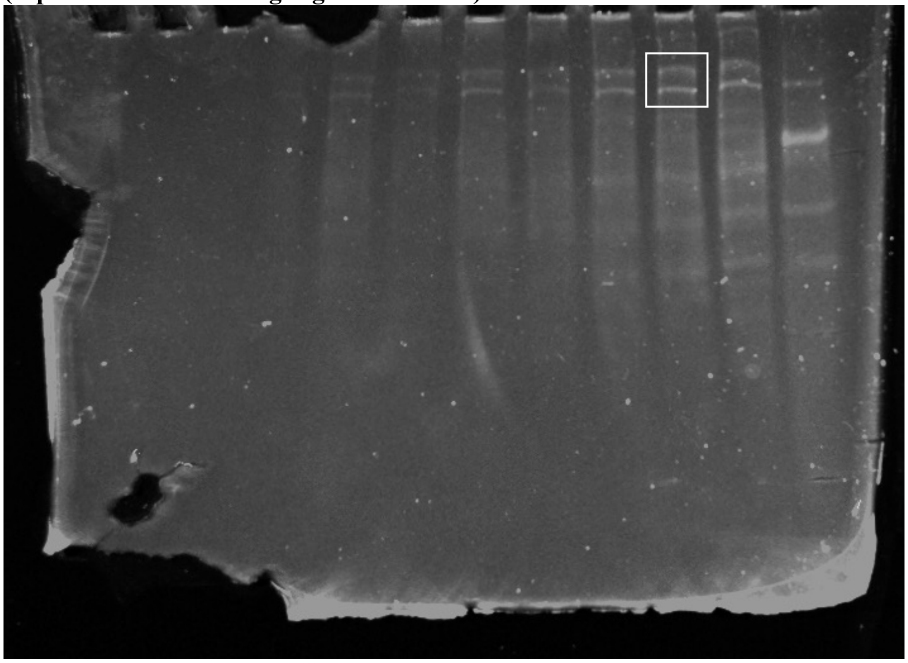

Lanes (1-7): Day3e, Day5d, Day7c, Day3d, Day1c, Day7b*, Day7a
